# Supplementary material for: Hsp22 with an N-Terminal Domain Truncation Mediates a Reduction in Tau Protein Levels
Source: Int J Mol Sci. 2020 Jul 30;21(15):5442. doi: 10.3390/ijms21155442 (PMC7432035; doi:10.3390/ijms21155442)
Supplement: Supplementary file 1 [file ijms-21-05442-s001.pdf]

Supplementary Data

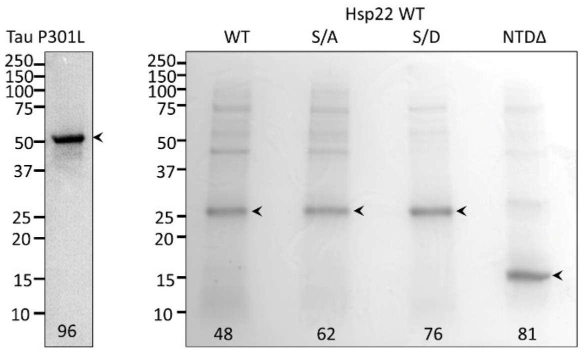

Figure S1: Coomassie stained gels of purified recombinant proteins used in ThT assays. Arrowheads identify bands at the anticipated size for each desired recombinant protein. Percent purity was estimated using Fiji/image J software and is labelled at the bottom of each lane.

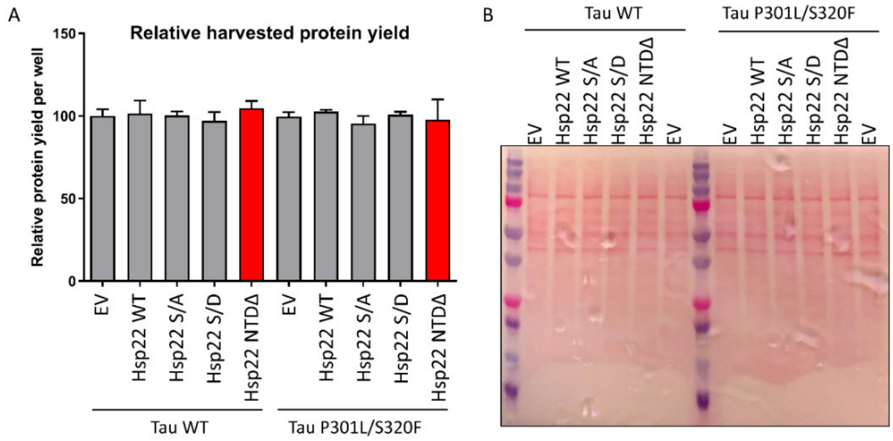

Figure S2: Relative total protein harvested and relative protein loading for SDS-PAGE and western blots with HEK293T cell lysates. (A) Cleared HEK293T cell lysates were analyzed for protein concentration to determine whether equivalent levels of total protein were harvested from each well. Conditions that repress cell growth or induce toxicity would be expected to decrease the total protein yield per well. Hsp22 NTDΔ samples are highlighted in red. Mean relative protein yield ± SEM are shown, n=3. The mean protein yield from three independent experiments was 371μg, 332μg, and 279 μg. For each experiment, protein concentration was normalized to the average yield for that experiment. (B) Ponceau S staining of a representative PVDF membrane after transfer from an SDS-PAGE gel. The image shows equivalent loading and transfer of total protein for these assays.

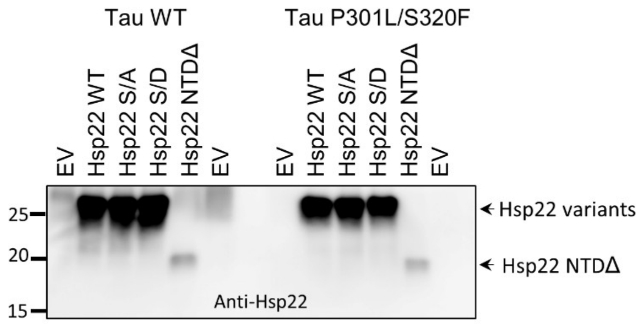

Figure S3. Confirmation of Hsp22 NTDΔ expression in transfected HEK293T cells. Triton-Soluble fractions from HEK293T cells co-transfected with plasmids as indicated, were evaluated by Western blot, as shown in Figure 5, except that cells were lysed in a smaller volume, more protein was loaded into each well (20 μg) and a long chemiluminescence exposure was captured to maximize sensitivity in order to visualize Hsp22 NTDΔ protein expression.

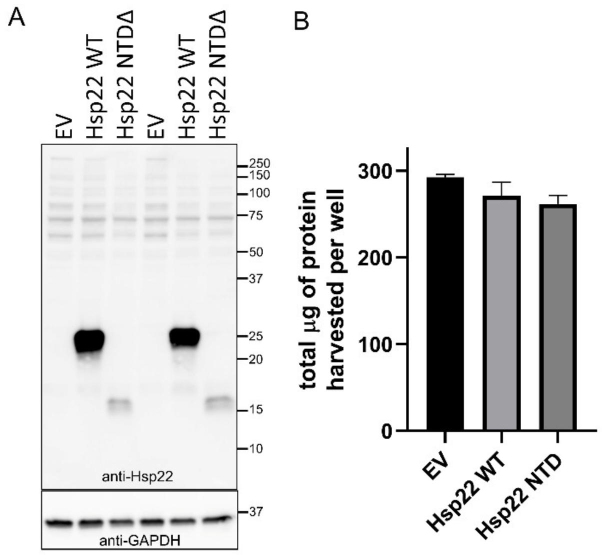

Figure S4: Exogenous Hsp22 WT and Hsp22 NTDΔ levels were compared 48 hours after transfection of HEK293T cells. (A) Western blot demonstrating relative levels of Hsp22 and GAPDH. Even in the absence of tau co-transfection, Hsp22 NTDΔ levels are much less than Hsp22 WT. Duplicate samples shown reflect separate transfected wells of cells harvested independently. (B) Total harvested protein from each well was measured. Mean with range bars are shown (n=2).
